# Bringing Greater Precision to Interactions Between Community Health Workers and Households to Improve Maternal and Newborn Health Outcomes in India

**DOI:** 10.9745/GHSP-D-20-00027

**Published:** 2020-09-30

**Authors:** Peter Smittenaar, B.M. Ramesh, Mokshada Jain, James Blanchard, Hannah Kemp, Elisabeth Engl, Shajy Isac, John Anthony, Ravi Prakash, Vikas Gothalwal, Vasanthakumar Namasivayam, Pankaj Kumar, Sema K. Sgaier

**Affiliations:** a Surgo Foundation, Washington, DC, USA.; b Centre for Global Public Health, Department of Community Health Sciences, University of Manitoba, Winnipeg, Canada.; c India Health Action Trust, Lucknow, Uttar Pradesh, India.; d National Health Mission, Government of Uttar Pradesh, Lucknow, Uttar Pradesh, India.; e Department of Global Health & Population, Harvard T.H. Chan School of Public Health, Boston, MA, USA.; f Department of Global Health, University of Washington, Seattle, WA, USA.

## Abstract

**Introduction:**

Community health workers (CHWs) play a key role in the health of mothers and newborns in low- and middle-income countries. However, it remains unclear by what actions and messages CHWs enable good outcomes and respectful care.

**Methods:**

We collected a uniquely linked set of questions on behaviors, beliefs, and care pathways from recently delivered women (n=5,469), their husbands (n=3,064), mothers-in-law (n=3,626), and CHWs (accredited social health activists; n=1,052) in Uttar Pradesh, India. We used logistic regression to study associations between CHW actions and household behaviors during antenatal, delivery, and postnatal periods.

**Results:**

Pregnant women who were visited earlier in pregnancy and who received multiple visits were more likely to perform recommended health behaviors including attending multiple checkups, consuming iron and folic acid tablets, and delivering in a health facility (ID), compared to women visited later or receiving fewer visits, respectively. Counseling the woman was associated with higher likelihood of attending 3+ checkups and consuming 100+ iron and folic acid tablets, whereas counseling the husband and mother-in-law was associated with higher rates of ID. Certain behavior change messages, such as the danger of complications, were associated with more checkups and ID, but were only used by 50%–80% of CHWs. During delivery, 57% of women had the CHW present, and their presence was associated with respectful care, early initiation of breastfeeding, and exclusive breastfeeding, but not with delayed bathing or clean cord care. The newborn was less likely to receive delayed bathing if the CHW incorrectly believed that newborns could be bathed soon after birth (which is believed by 30% of CHWs). CHW presence was associated with health behaviors more strongly for home than facility deliveries. Home visits after delivery were associated with higher rates of clean cord care and exclusive breastfeeding. Counseling the mother-in-law (but not the husband or woman) was associated with exclusive breastfeeding.

**Conclusion:**

We identified potential ways in which CHW impact could be improved, specifically by emphasizing the importance of home visits, which household members are targeted during these visits, and what messages are shared. Achieving this change will require training CHWs in counseling and behavior change and providing supervision and modern tools such as apps that can help the CHW keep track of her beneficiaries, suggest behavior change strategies, and direct attention to households that stand to gain the most from support.

## Abstract (precis)

We identified how the quantity and quality of actions taken by community health workers can be refined to move from a one-size-fits-all model to a precision approach that stands to benefit the health of the mothers and newborns they support.

## Teaser

**Key Findings:**

- Community health worker (CHW) presence, number and timing of visits, behavior change messaging strategies, and focus on specific household members for different behaviors associates with maternal and newborn care practices.
- Local sociocultural factors such as the decision dynamics of households and common false beliefs about neonatal care should inform how the CHW communicates.

**Key Implications:**

- Program managers can use these insights to adapt the CHW training, incentives, and tools to achieve greater impact.
- Other CHW programs can use this approach to identify opportunities to improve the effectiveness of their workers in terms of communication, knowledge, and number and timing of visits.

## INTRODUCTION

In low- and middle-income countries, community health workers (CHWs) provide basic but lifesaving support for those who have little access to formal health care, especially for reproductive, maternal, neonatal, and child health services.^1^ Their impact on health outcomes has been documented^1^^–^^4^ and demonstrates considerable further potential.^5^^,^^6^

One barrier to realizing this potential is a lack of understanding about why CHWs are effective in achieving some health outcomes, but less so in others. Most studies of CHWs are descriptive, for example, capturing CHWs’ level of clinical knowledge or the number of visits they make.^7^ Explan-atory studies of the mechanisms by which CHWs achieve impact (or fail to) are needed. Such insights can be used to guide the development of CHW programs.

We report a detailed quantitative study of the interactions between household members and their CHW during pregnancy, delivery, and early postnatal care. The CHWs are part of India’s accredited social health activist (ASHA) program—the world’s largest CHW program, which has recruited about 1 million volunteers. ASHAs are trained by the government’s National Rural Health Mission and receive financial incentives for their work. Each ASHA is responsible for guiding about 1,000 people in her community through interactions with health services, providing basic medical and contraceptive supplies, and educating them on basic health topics.^8^ In practice, ASHAs divide their time between accompanying people (primarily women) to health facilities, documenting their work, supporting regular village health and nutrition events, and visiting families in their homes.

A recent review of research on the ASHA program suggested an overall positive impact, but most studies showed mixed results in terms of performance and outcomes.^7^ This is in part explained by the constraints of the health system in which the ASHA operates,^9^ but also by the complexities of navigating decision dynamics in rural Indian households. Upon marrying, most women move in with their husband’s family, meaning that other family members often play decisive roles in certain health-related decisions.^10^ In Uttar Pradesh, where this study was performed, women rarely make maternal and child health decisions on their own. Rather, the husband and mother-in-law (MIL) play a major role.^11^ The husband’s knowledge about ANC practices and delivering in a health facility and the MIL’s knowledge about early initiation of breastfeeding (EIBF) practices are associated with whether the household performs these practices.^12^^,^^13^ Our qualitative work suggests men are most likely to be involved when decisions have financial repercussions, such as whether to deliver in a facility, whereas the MIL is deferred to for decisions around home care, such as breastfeeding or cord care (unpublished results). Social norms further impinge on these decisions, with norms themselves constantly evolving. Altogether, this leaves the ASHA with difficult decisions on who to target in the household to effect behavior change toward recommended health behaviors, and the optimal target is not necessarily the primary decision maker if this individual is not receptive to ASHA advice.

Rural households often have complex decision dynamics for certain health-related decisions.

We designed a novel approach to study the mechanisms of CHW impact around pregnancy, childbirth, and early postnatal care. We collected detailed questionnaire data from recently delivered women, their husbands, MILs, and ASHAs. Understanding perspectives of each of the decision makers in the household allowed us to identify ways to make ASHA home visits more impactful. We set out to answer the following questions: (1) What are the rates of recommended behaviors? (2) What support are women receiving from their ASHA? (3) Is there an association between the number and timing of ASHA home visits or ASHA’s presence at birth and uptake of recommended health behaviors? (4) What types of behavior change messages are favored by ASHAs during the antenatal period, and is household behavior associated with favored messages? (5) To what extent is uptake of recommended behavior associated with whether the woman, husband, and mother-in-law are counseled by the ASHA? For this last question, we did not have strong hypotheses as to whom the ASHA should target, given that impact is a function of the ASHA’s ability to talk to the respective household member, receptiveness to advice by the recipient, and the recipient’s power in the household. We also examine the extent to which ASHA presence at birth is associated with respectful care, which is often compromised in low-resource settings.^14^

Understanding perspectives of the household decision makers helped to identify ways to make CHW home visits have more impact.

Our goal is to stimulate reflection on the impact of a particular cadre of CHWs, especially how such impact is achieved, to generate lessons that may be relevant for other CHW programs with regard to training, supervision, and tools.

## METHODS

### Sampling Household Members and CHWs

The primary sampling unit was the ASHA catchment area, which usually comprises 1 village. Of all rural catchment areas in India’s Uttar Pradesh state (UP), 1,575 were selected pseudorandomly, with higher likelihood of sampling assigned to those previously identified by the UP government as high-priority for interventions. The final sample of 1,575 areas had an average population of 1,064 (standard deviation [SD]=344) people as reported by the ASHA. All households with a living woman who had given birth in the past 2 months (including perinatal death cases) were approached for the survey. Between September 2017 and January 2018, 6,078 interviews of women were conducted across 1,514 ASHA catchment areas (no recently delivered women were identified in 61 catchment areas). Of these, 609 interviews were excluded because they were conducted in the first week after birth (thus did not yet have data on their postnatal behaviors), the women declined to consent, they were duplicates of later interviews (e.g., if the interview was initiated but then rescheduled), or they were discontinued midway through the survey. The final sample analyzed was 5,469 women across 1,499 catchment areas (76 of 1,575 catchment areas had no women included and were therefore excluded).

One ASHA was assigned to each catchment area. For most (n=5,278) of the final sample of 5,469 women, their ASHA was also surveyed if the catchment area had an ASHA assigned and she could be contacted (of 1,575 catchment areas, 1,502 ASHAs completed an interview, of whom 1,435 had at least 1 woman from their area interviewed). This approach allowed us to understand how traits, beliefs, experiences, behaviors, and other characteristics of the ASHA and woman were associated at the individual level.

Data were collected through face-to-face interviews at the homes of women and ASHAs. Details of the questionnaires, including design and content, are described in Supplement 1. Each respondent provided informed oral consent at the time of screening and written consent before the full interview. Interviewers were trained on sensitive topics and ethics of conducting interviews. The study was approved by Sigma Institutional Re-view Board (approval number 10032/IRB/D/16-17, New Delhi, India).

#### Public Involvement

There was no public involvement element. Before launching the main study, we conducted a pilot test where we interviewed a handful of recently delivered women, their husbands, MILs and ASHAs to learn from their experiences before, during, and after childbirth. Written consent was obtained and the information provided during the interview was treated as confidential. Based on the pilot, we refined the survey to make it simpler for both interviewers and respondents. The public was not further involved in the study nor will they be involved in dissemination of the results, which will be shared with health programs and the government.

### Variable Selection and Statistical Analysis

We aimed to cover key actions taken by the ASHA, as well as key health behaviors and outcomes that are important for households. From the household survey, we selected 9 outcomes known to be important for maternal and neonatal health^15^^,^^16^: (1) attending 3 or more antenatal checkups at a monthly village health and nutrition day or at a medical facility; (2) taking at least 100 iron and folic acid (IFA) tablets over the course of pregnancy; (3) having an institutional delivery (ID) at a public or private facility; (4) receiving respectful care with no experience of physical or verbal mistreatment from staff; (5) staying in the facility for at least 24 hours after delivery for women without cesarean delivery only; (6) initiating breastfeeding within 1 hour of birth (EIBF) (for women without cesarean delivery only); (7) promoting exclusive breastfeeding (EBF); (8) applying nothing to the umbilical cord stump that might cause infection (clean cord care); and (9) delaying bathing of the baby by at least 72 hours. Although the 72-hour delay is longer than usually selected in studies,^15^ it is the period advised by the UP government.

To understand decision making dynamics, we collected perception, knowledge, and attitudinal data, linking women’s responses to responses by their husbands, mothers-in-law, and health workers.

We also defined 8 ASHA actions, as reported by the recently delivered woman, that had the potential to impact women’s health behaviors^16^: (1) whether any antenatal home visit was made; (2) number of antenatal home visits (for the subgroup with 1+ visits only); (3) month of pregnancy of the first home visit (for the subgroup with 1+ visits only); (4) whether the ASHA was present for any part of delivery, irrespective of the place of delivery; (5) how many hours the ASHA was present at delivery (for the subgroup that had an ASHA present only); (6) how many hours the ASHA was present immediately after birth minus the hours present before birth (for the subgroup with an ASHA present only); (7) whether any postnatal visit was made in the first week after birth; and (8) number of postnatal home visits in the first week after birth (for the subgroup with 1+ postnatal visits only).

### Statistical Analysis: Logistic Regression

We used logistic regression to test for associations between ASHA actions and household behaviors, both as reported by the household. Each action-outcome association had its own specific set of control variables as well as a particular (sub)group of participants included, so a separate regression was performed for each association (Supplement 2 includes discussion of multiple comparisons). Nonetheless, each regression presented here included a set of demographic control variables captured from the woman (Supplement 3, Table): religion, caste, parity, years of education, age of woman, electricity available in house as proxy for wealth, and household type (nuclear versus multigenerational). The control variables were selected based on authors’ knowledge of the context, existing literature, and availability in the survey.

We implemented unweighted regressions in the R software package.^17^ To visualize the relevant relationships in graphs, we transformed the predictors to a categorical variable and performed the regressions again so we could calculate estimated marginal proportions corrected for all covariates.^18^ No adjustments were made for clustering of the data, as every ASHA on average only covered 3–4 recently delivered women in the survey sample.

We identified each ASHA’s “preferred message” for convincing women to attend checkups and go for ID, respectively, from the ASHA survey (Supplement 1). Household members and ASHAs were linked by a unique ASHA catchment identifier that was recorded in both surveys. We then regressed each woman’s attendance of 3+ checkups and ID, respectively, onto the preferred message of the ASHA in their catchment area. To partially control for features of the ASHA that might correlate with their preferred message, we not only used the set of covariates used for the main analyses but also added covariates derived from the ASHA questionnaire: demographics, working hours, tenure, earnings, and number of familiar messaging strategies.

## RESULTS

The demographics of the women, husbands, and MILs are described in Supplement 3.

### Rates of Recommended Health Behaviors Fell Below Target

We compared observed rates to target rates set by the government for the end of 2019, where available (Technical Support Unit, Uttar Pradesh, Phase 2 targets). Percentages were weighted for oversampling of government interest areas to ensure they are representative of the state of Uttar Pradesh, but n were not (denominator varied in part due to respondents could answer they did not remember what precisely happened). Only 49% (2,625/5,467) of pregnant women reported attending 3+ checkups (no target available), 14% (782/5,469) reported having taken the recommended 100 IFA tablets (target: 50%), 82% (4,428/5,469) of women had an ID (target: 80%), and 21% (817/3,898) of women reported having remained in hospital for at least 24 hours of birth (target: 80% stay for 48+ hours). In the postnatal care phase, 58% (2,823/4,925) of women reported EIBF (target: 80%); 63% (3,453/5,469) EBF (target: 80% for full 6 months); 51% (2,554/5,221) reported having first bathed their newborns 72+ hours after delivery (target: 90%); and 18% (915/5268) reported not applying anything to the umbilical cord stump of their newborn (target: 90%).

### ASHA Support Varied Across the Maternal and Newborn Care Pathway

**FIGURE 1.**
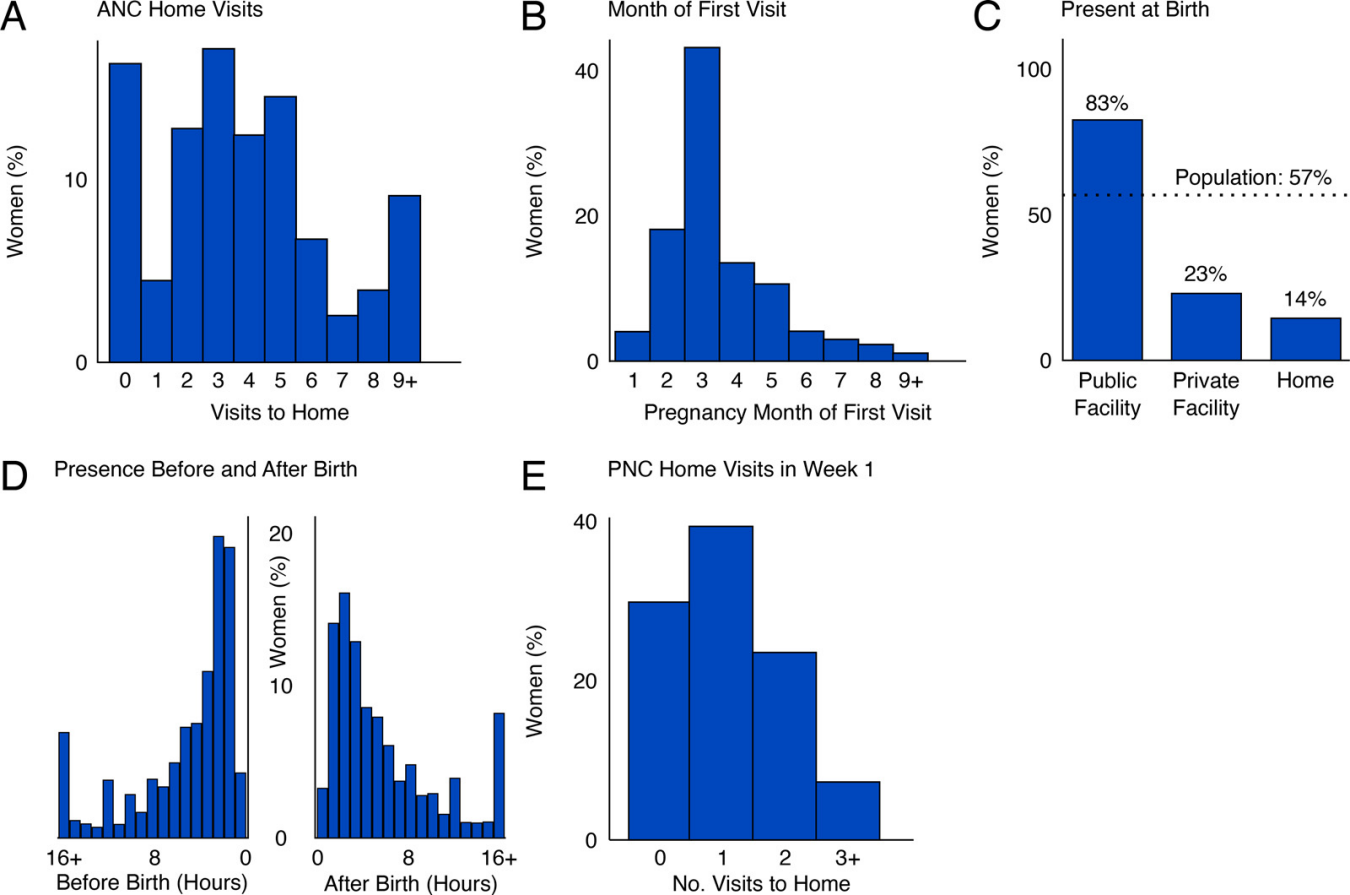
(A) Number of CHW Home Visits to Each Woman^a^; (B) Month of Pregnancy in Which First Visit Was Received^b^; (C) Rate of CHW Presence at Birth^c^; (D) Distribution of Hours Present Before and After Delivery^d^; (E) Number of CHW Home Visits in the First Week After Delivery^e^ Abbreviations: ANC, antenatal care; CHW, community health worker; PNC, postnatal care.Each y-axis represents the percentage of women receiving a particular level of service from their CHW, weighted to be representative of rural Uttar Pradesh.^a^ N=5,469 women.^b^ For the 84% of women that received at least 1 visit (n=4,541 women).^c^ Dashed line shows the average across all births (n=5,469 women).^d^ For the 57% of women that had the CHW present for any part of delivery, hours present as recollected by the woman (n=3,071).^e^ n=5,383 women.

The ASHA is expected to visit each woman at least 3 times during pregnancy. Most households (84%) reported at least 1 such visit (Figure 1A). For households receiving 1 visit, the ASHA visited relatively often (median 4 times across pregnancy) and early in pregnancy (at month 3.5 on average (Figure 1B); guidelines recommend the ASHA visit as soon as the woman is aware of the pregnancy. When the woman went into labor, the ASHA was incentivized to accompany her to a public facility or be present for home birth. The ASHA was present for 83% of deliveries that happened in a public facility (Figure 1C). However, the ASHA was largely absent from deliveries at home (present for 14% of deliveries) and in private facilities (present for 23% of deliveries); overall 57% of women reported that the ASHA was present at the delivery (Figure 1C). When the ASHA was present for delivery, she would often be there for several hours before and several hours after delivery (Figure 1D). However, sometimes the ASHAs were present for 16+ hours before delivery (7% of cases where ASHA was present) or 16+ hours after delivery (8% of cases where ASHA was present [Figure 1D]). Only 31% of women reported having received 2 visits or more in the first week after birth, and 30% reported having received no home visit at all over the first week after birth.

### ASHA Work Hours Were Skewed Toward Hospital Visits and Paperwork

Time expenditure was determined by asking the ASHA how frequently she performed a particular task (e.g., “accompanied woman to hospital” or “performed census”), and how long it took on average. Adding up her reproductive, maternal, and neonatal health activities and related administrative tasks (but excluding additional duties on other health topics), the average ASHA reported 65.7 hours per month on this work (Supplement 4, Supplemental Figure 1 has a visual representation). Her monthly work consisted of:

- Accompanying women to hospital for delivery or checkups (22 hours; average ASHA spent 33.5% of her time on this task)
- Documentation (19 hours; 28.9%)
- Attending Village Health and Nutrition Day (6.2 hours; 9.4%)
- Home visits to women after birth (5.8 hours; 8.8%)
- Home visits to pregnant women (4.2 hours; 6.4%)
- Home visits to couples eligible for family planning (3.4 hours; 5.2%)
- Village population census (2.6 hours; 4.0%)
- Home visits for child immunization (2.5 hours; 3.8%)

This showed that the bulk of the ASHA’s time was spent on accompanying women on visits to health facilities and on paperwork, with home visits comprising only 24.2% of her time spent on maternal, neonatal, and child health.

### Health Behaviors Were Associated With Timing and Number of ASHA Visits and Preferred Messaging

#### ASHA Support to Pregnant Women

##### Quantity and Timing of Antenatal Home Visits Was Associated With Health Behaviors

**FIGURE 2.**
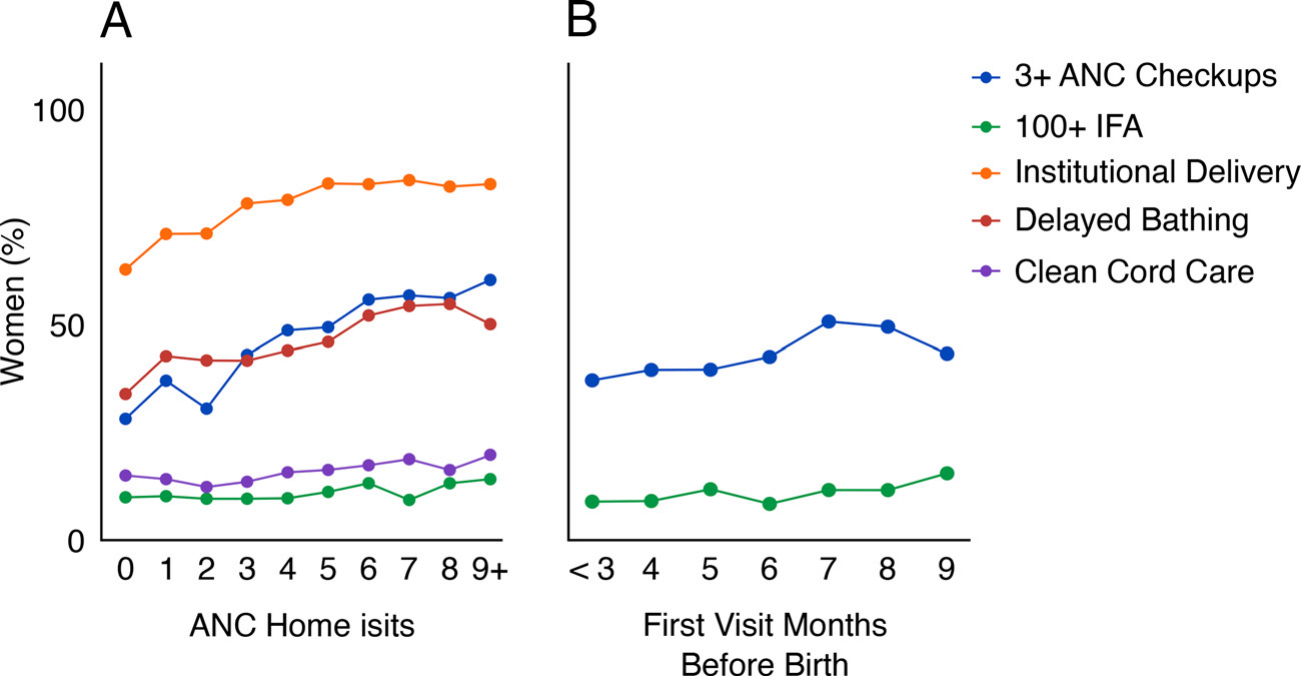
Estimated Relationships Between Health Behaviors and (A) Number of CHW Home Visits and (B) Timing of First Home Visit Abbreviations: ANC, antenatal care; CHW, community health worker; IFA, iron and folic acid.Only significant associations are shown, see Table 1 for all associations. All means are estimated marginal proportions adjusted for covariates.^a^ n=5,438 women.^b^ n=4,541 women, excluding those who received no visit.

Antenatal home visits had substantial positive associations with health behaviors (Table and Figure 2A). Receiving at least 1 visit was strongly associated with attending 3+ checkups (adjusted odds ratio [aOR]=2.22; 95% confidence interval [CI]=1.90, 2.59), ID (aOR=2.20; 95% CI=1.85, 2.61), and delayed bathing (aOR=1.55; 95% CI=1.30, 1.86). More visits further increased these odds (a positive “dose-response” effect, though we cannot say whether the home visits preceded check-ups or IFA consumption), with the benefit of additional visits tapering off around 4–6 visits: (Figure 2A; 3+ checkups aOR=1.14; 95% CI=1.11, 1.17); ID (aOR=1.09; 95% CI=1.05, 1.14); and delayed bathing (aOR=1.08; 95% CI=1.04, 1.11). Number of home visits was also associated with IFA consumption (aOR=1.05; 95% CI=1.01, 1.09) and cord care (aOR=1.07; 95% CI=1.03, 1.12), though these associations were relatively weak (Figure 2A). In contrast, number of antenatal home visits was not associated with duration of facility stay after birth (aOR for receiving at least 1 visit=0.98; 95% CI=0.78, 1.23), EBF (aOR=0.99; 95% CI=0.84, 1.18), and only weakly if at all with EIBF (aOR=1.21; 95% CI=1.00, 1.45).

**TABLE.** Regression Results for CHW Actions in Uttar Pradesh, India.

CHW action	Any Antenatal Home Visit	Number of Antenatal HomeVisits^a^	Timing of First Antenatal Home Visit^a^	Present for Any Part of Delivery	Hours Present (Before + After)^b^	Pre- vs Postpartum Hours (After - Before)^b^	Any Home Visit in First Week After Delivery	Number of Home Visits in First Week After Delivery^c^
**Adjusted Odds Ratio**^d^ **(95% CI); *P* value**								
Antenatal								
Received 3+ checkups	2.22 (1.90, 2.59) *P*<.001	1.14 (1.11, 1.17) *P*<.001	1.10 (1.06, 1.15) *P*<.001					
Consumed 100+ IFA	1.11 (0.9, 1.37) *P*=.35	1.05 (1.01, 1.09) *P*=.007	1.07 (1.01, 1.13) *P*=.03					
Delivery								
Delivered in facility	2.20 (1.85, 2.61) *P*<.001	1.09 (1.05, 1.14) *P*<.001	1.05 (0.99, 1.10) *P*=.08					
Respectful care				1.55 (1.14, 2.07) *P*=.004	1.01 (0.99, 1.03) *P*=.31	1.01 (0.99, 1.04) *P*=.29		
Stayed at facility for 24+ hours	0.98 (0.78, 1.23) *P*=.84	1.01 (0.97, 1.05) *P*=.72		1.02 (0.82, 1.27) *P*=.84				
Postnatal								
Early initiation breastfeeding	1.21 (1.00, 1.45) *P*=.04	0.99 (0.96, 1.02) *P*=.59		1.32 (1.12, 1.56) *P*=.001	1.00 (0.99, 1.01) *P*=.84	1.00 (0.98, 1.01) *P*=.60		
Delayed bathing 72+ hours	1.55 (1.30, 1.86) *P*<.001	1.08 (1.04, 1.11) *P*<.001		0.95 (0.81, 1.12) *P*=.57	1.00 (1.00, 1.01) *P*=.76	1.01 (0.99, 1.02) *P*=.31		
Exclusively breastfed	0.99 (0.84, 1.18) *P*=.95	1.01 (0.98, 1.05) *P*=.39		1.24 (1.06, 1.45) *P*=.008	1.00 (0.99, 1.00) *P*=.46	1.01 (0.99, 1.02) *P*=.37	1.44 (1.25, 1.66) *P*<.001	1.21 (1.08, 1.36) *P*=0.001
Clean cord care	1.00 (0.78, 1.29) *P*=.99	1.07 (1.03, 1.12) *P*<.001		1.19 (0.96, 1.49) *P*=.11	1.00 (1.00, 1.01) *P*=.12	0.99 (0.98 1.01) *P*=.43	1.40 (1.14, 1.73) *P*=.002	1.56 (1.37, 1.77) *P*<.001

Abbreviations: CHW, community health worker; CI, confidence interval; IFA, iron and folic acid.

a Women with 1+ antenatal home visit only.

b Women with community health worker presence only.

c Women with 1+ postnatal home visit within first week after birth only.

d An adjusted odds ratio (aOR) greater than 1 means the household behavior was more likely. Each aOR is from a separate regression with appropriate covariates depending on the relationship being estimated, including corrections for demographics, parity, location of delivery, and other CHW actions.

We observed that earlier first visits by the ASHA increased the likelihood that the woman attended 3+ checkups (aOR=1.10; 95% CI=1.06, 1.15) and consumed 100+ IFA tablets (aOR=1.07; 95% CI=1.01, 1.13) (Table and Figure 2B; included a covariate for total number of antenatal home visits). However, no significant association was found with ID (aOR=1.05; 95% CI=0.99, 1.10) (Table).

##### Targeting Advice to the Right Decision Maker Mattered During Antenatal Visits

**FIGURE 3.**
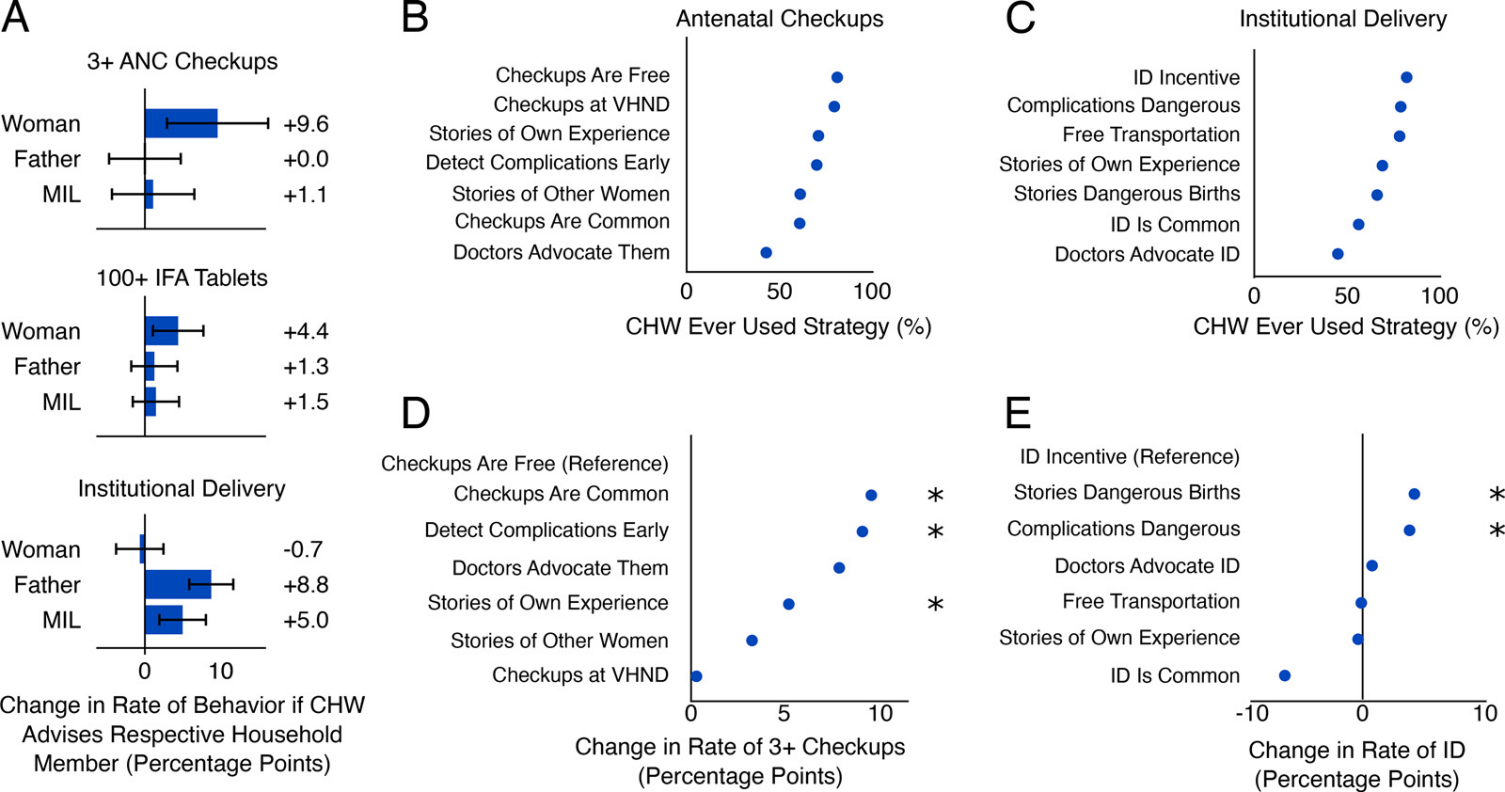
(A) Increase in Health Behavior if Household Member Reported Having Talked to CHW About the Particular Behavior^a^; (B) Percentage of CHW Workforce That Has Ever Used Each Type of Message When Convincing Households to Attend Checkups^b^; (C) Percentage of CHW Workforce That Has Ever Used Each Type of Message When Convincing Households to Deliver in Facility^c^; (D) Predicting Whether a Woman Attended 3+ Checkups Based on the Preferred Behavior Change Message of the CHW^d^; (E) Predicting Whether a Woman Had an ID Based on the Preferred Behavior Change Message of the CHW^e^ Abbreviations: ANC, antenatal care; CHW, community health worker; ID, institutional delivery; IFA, iron and folic acid; MIL, mother-in-law; VHND, village health nutrition day.All rates represent adjusted means, including covariates for several indicators of CHW quality and performance. * Indicates *P*<.05 compared to the reference.^a^ Expressed as absolute increase with 95% confidence intervals (percentage points) based on regression. n=2,057 households that received CHW visit and had woman, husband, and MIL interviewed.^b^ Self-reported in the CHW survey. n=1,318 CHWs that had at least 1 household receiving a visit from them in the data.^c^ n=1,318 CHWs.^d^ The most commonly preferred message is used as reference, and differences represent absolute changes in the rate of behavior. n=4,134 women.^e^ N=4135 women.

To better understand the mechanisms underlying the associations from Figure 2, we analyzed the targeting of household members with messages. The recently delivered woman, her husband, and her MIL each reported whether the ASHA advised them on checkups, IFA, and ID, respectively. We tested whether such advice was associated with behavior, noting that for behavior to change the ASHA needed to know how to communicate well to each particular household member and the recipient needed to have the required influence in the household. Attendance of 3+ checkups was more likely in households where the ASHA had advised the woman on antenatal checkups (Figure 3A): aOR for counseling of mother (independent variable) on 3+ checkups (dependent variable)=1.47; 95% CI=1.12, 1.93). Similarly, consumption of 100+ IFA tablets was more likely in households where the ASHA had counseled the mother on IFA tablets; women were equally likely to receive these tablets from either an ASHA or auxiliary nurse midwife (Figure 3A; aOR=1.48; 95% CI=1.08, 2.05). Counseling the husband or MIL on checkups or IFA, respectively, had no association with likelihood of performing such behaviors (Figure 3A). In contrast, advice to go for ID was associated with increased rates of ID only if the husband (aOR=2.16; 95% CI=1.66, 2.83; *P*<.001) and MIL (aOR=1.52; 95% CI=1.18, 1.95; *P*=.001) received it (Figure 3A). For each of the 3 behaviors, the pregnant woman was most likely to report having been counseled, followed by the MIL, and finally the husband (Supplemental Figure 2, Supplement 5).

Each health behavior had its own set of decision makers to target by the ASHA.

##### ASHAs Lacked Awareness of Effective Behavior Change Messages

To investigate the type of message most likely to be associated with a target behavior, we asked all ASHAs what messages they used when trying to convince families to go for checkups and ID (Figure 3B–C). Financial arguments were most common (e.g., “Checkups are free”), whereas social norm arguments (e.g., “It’s common practice”) were least often reported. For each type of message, 50%–80% of ASHAs reported using them.

The most commonly reported message types were not necessarily those most strongly associated with the target behaviors. The 2 least-reported message types for checkups—saying that checkups were now common and that doctors recommended them—were associated with the highest rate of checkup attendance (Figure 3D; *P* values against reference of “checkups are free”: checkups are common, *P*=.05; doctors advocate checkups, *P*=.08; detected complications early, *P*<.001; stories of own experience, *P*=.04; all other *P*>.10). Conversely, the commonly reported message that “checkups are free” was associated with the lowest rate of 3+ checkups. For ID, messages about complications were most strongly associated with ID, whereas the least reported “ID is common” was associated with the lowest rates of ID (Figure 3E; *P* values against reference of “ID incentive”: complications are dangerous, *P*=.02; stories of dangerous births, *P*=.02; ID is common, *P*=.07; all other *P*≥0.1).

Most CHWs could greatly benefit from a more expanded repertoire of behavior change messages.

#### ASHA Presence at Birth

##### ASHA Presence Was Associated With EIBF and EBF

**FIGURE 4.**
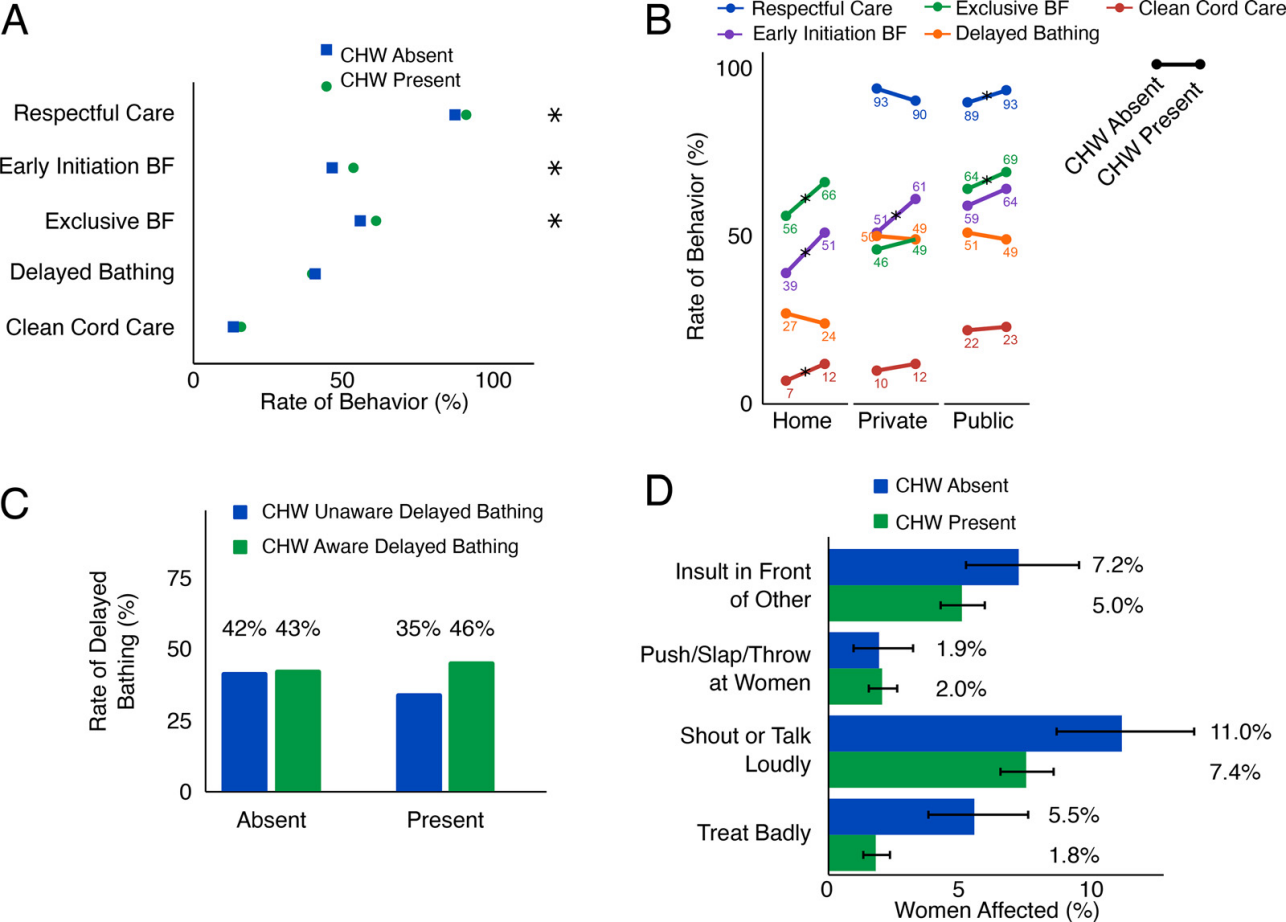
(A) Relationship Between Outcomes and CHW Presence at Birth Irrespective of Location of Delivery^a^; (B) Relationship Between Outcomes and CHW Presence at Birth by Location of Delivery^b^; (C) Association of CHW’s Awareness of Delayed Bathing and Practice of Delayed Bathing When CHW Was Absent or Present^c^; (D) Rate of Different Forms of Poor Treatment at Public Facility Deliveries When the CHW Was Absent or Present^d^ Abbreviations: BF, breastfeeding; CHW, community health worker.^a^ N=5,240 women. * Indicates statistical significance at *P*<.05.^b^ N=984 women at home, 1068 at private, 3123 at public.^c^ N=4831 women.^d^ N=3244 women. Error bars represent 95% CI.

ASHAs encouraged families to go to a hospital for birth and often would stay for hours (Figure 1D). ASHA presence at delivery was associated with a 7-percentage-point higher likelihood of EIBF (aOR=1.32; 95% CI=1.12, 1.56) and a 6-percentage-point higher likelihood of EBF (aOR=1.24; 95% CI=1.06, 1.45) (Table and Figure 4). Although women generally reported high levels of respectful care by staff, ASHA presence during delivery was associated with a 3.6-percentage-point higher likelihood of women receiving respectful care (aOR=1.55; 95% CI=1.14, 2.07) (Figures 4A and 4D). ASHA presence was not found to be associated with clean cord care (aOR=1.19; 95% CI=0.96, 1.49) or delayed bathing (aOR=0.95; 95% CI=0.81, 1.12). We also did not observe an association between the duration of ASHA presence and any of the 4 postnatal care behavior outcomes or between her presence before versus after birth and any of the outcomes (see Table for aOR values).

##### ASHA Presence Had a Protective Effect for Home Deliveries

ASHA presence at home deliveries was associated with higher likelihood of EIBF (+12 percentage points, *P*=.01), EBF (+10 percentage points, *P*=.04), and clean cord care (+5 percentage points, *P*=.05). In contrast, at public facilities, the only significant association was with EBF (+5 percentage points, *P*=.04). At private facilities, the only significant association was with EIBF (+10 percentage points, *P*=.04). The association between ASHA presence and cord care was significantly greater for home deliveries than public (*P*=.03) or private (*P*=.04) deliveries. Similarly, respectful care was more likely when the CHW was present for public-facility deliveries compared to private-facility deliveries (*P*=.001). In contrast, none of the other behaviors (EBF, EIBF, or delayed bathing) showed a stronger association with ASHA presence at home compared with birth in facilities. These marginally significant findings suggest that ASHA presence at home births might be particularly impactful.

##### Incorrect ASHA Knowledge Was Associated with Early Bathing

Thirty percent of ASHAs incorrectly believe bathing immediately after birth did not put the newborn at risk. We found that if the ASHA had incorrect knowledge on delayed bathing, her presence at birth was associated with lower likelihood of delayed bathing (Figure 4C; presence-knowledge interaction aOR=2.07; 95% CI=1.33, 3.22; *P*<.001). The marginal proportions suggested that incorrect knowledge of ASHAs regarding recommended delayed bathing practices might be encouraging incorrect behaviors (Figure 4C).

Having a worker present with incorrect delayed bathing knowledge increases the risk of early bathing.

#### ASHA Actions in Postnatal Period

##### ASHA Visits Had a Protective Effect on Clean Cord Care and Exclusive Breastfeeding

**FIGURE 5.**
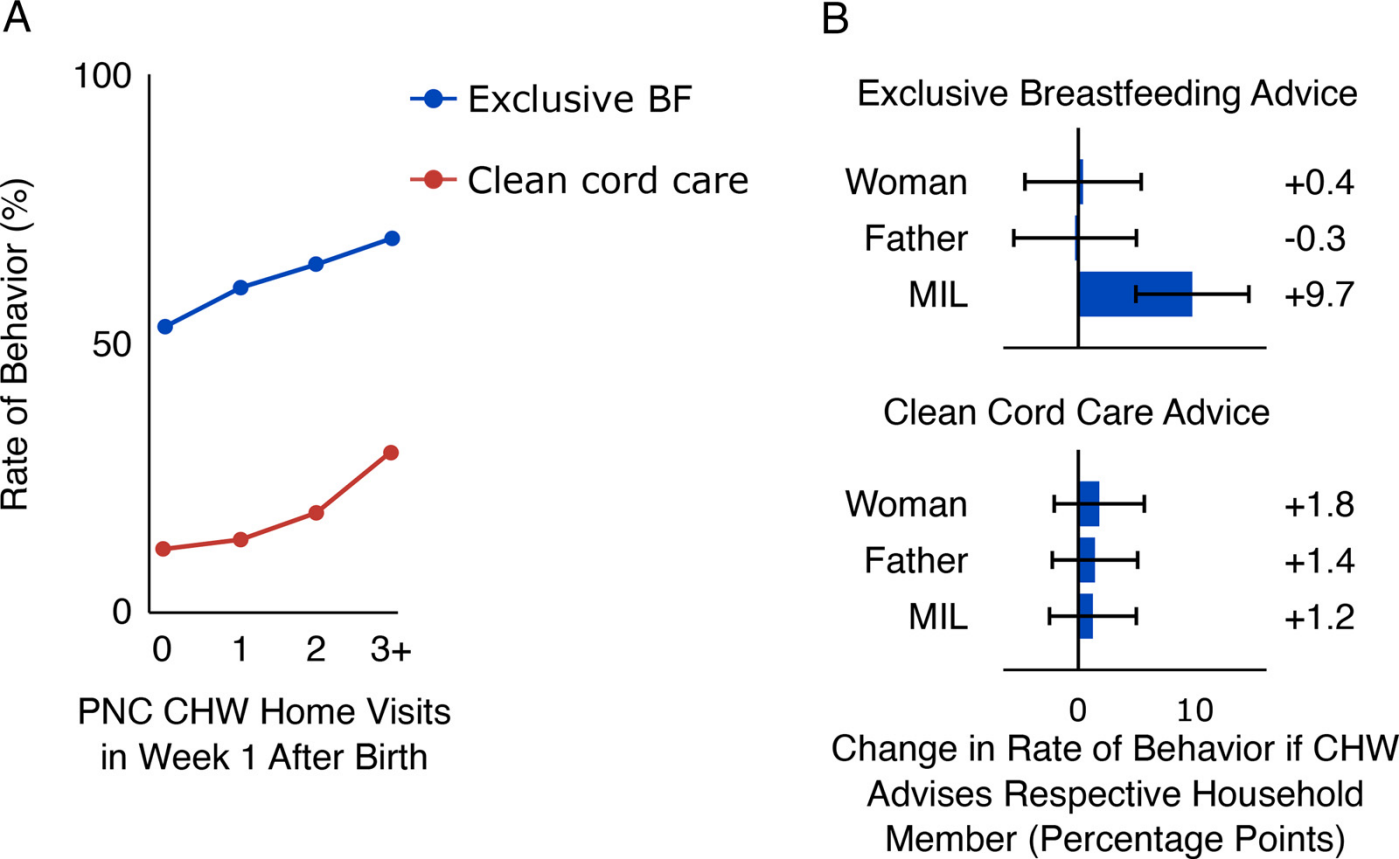
(A) Association of Number of PNC Visits With Rate of Exclusive Breastfeeding and Clean Cord Care^a^; (B) Increase in Health Behavior if Household Member Reported Having Talked to CHW About the Particular Behavior^b^ Abbreviations: BF, breastfeeding; CHW, community health worker; MIL, mother-in-law; PNC, postnatal care.^a^ N=5,185 women.^b^ N=2,057 households. Error bars represent 95% confidence interval.

Receiving 1 or more ASHA visits (compared to receiving none) in the first week after birth was associated with both higher likelihood of EBF (aOR=1.44; 95% CI=1.25, 1.66) and clean cord care (aOR=1.40; 95% CI=1.14, 1.73). For women reporting they had received at least 1 visit, additional visits further improved the odds of those behaviors: EBF (aOR=1.21; 95% CI=1.08, 1.36) and clean cord care (aOR=1.56; 95% CI=1.37, 1.77) (Table and Figure 5A). Those reporting 3+ visits had substantially better odds of performing recommended behaviors (Figure 5A).

##### Targeting MIL Positively Was Associated With Exclusive Breastfeeding

Advice from the ASHA to the MIL was associated with substantially higher rates of EBF (aOR=1.54; 95% CI=1.24, 1.92; *P*<.001) (Figure 5B, top), whereas the likelihood of clean cord care was unaffected by the ASHA’s advice (Figure 5B, bottom). The data were insufficiently granular to tease apart the relationship between timing of visits and when the decisions were actually made in the household. As with antenatal counseling, the recently delivered woman was most likely to report having been counseled, followed by the MIL and finally the husband (Supplemental Figure 2, Supplement 5).

## DISCUSSION

This study analyzed a large cross-sectional data set from women who had delivered a newborn in the preceding 2 months in UP, India, their husbands, MILs, and ASHAs. Having such rich data on each household allowed us to explore possible relationships between ASHA activities and target behaviors and gain insights to improve ASHA performance. As with earlier studies,^19^^–^^22^ we found that ASHA home visits were associated with a range of target behaviors. The study also offers insights into the influence of ASHA knowledge, timing of visits, messaging strategies, and decision-maker targeting on care practices. Such insights can enable program managers to further adapt the ASHA program to achieve greater impact. Other CHW programs can also learn from our findings when considering what guidance to give their workers on communication, knowledge, and number and timing of visits.

In the survey, two-thirds of pregnant women reported having received 3 antenatal home visits from their ASHA, though we observed better health behaviors up to 6 or 7 antenatal home visits. There are no guidelines for ASHAs on how many home visits to make and when,^23^ though the 2016 World Health Organization recommendation is that a woman attends 8 antenatal clinic visits during pregnancy.^16^ In resource-limited settings such as those in UP, CHW home visits might have to supplement a smaller number of clinic visits. In the first week after birth, only 31% of women receive 2+ visits, and we observed better health behaviors for up to 3+ postnatal visits in the first week after birth. ASHAs spend considerably less time visiting households than the 34–65 hours monthly suggested by guidelines.^23^ Therefore, it seems that increasing the number of home visits would be beneficial. Improving the content of each visit (e.g., through stronger communication strategies) might help ASHAs achieve behavior change more readily, thus reducing the need for additional home visits. Either way, we recommend clearly communicating a guideline for the number of antenatal home visits ASHAs should make, which we were unable to identify in ASHA training materials. We also note the absence of a relationship between antenatal home visits and recommended breastfeeding behaviors. We have no data on counseling about postnatal care during antenatal home visits, but counseling on EIBF, EBF, and clean cord care—to both the woman and her MIL—should be a priority in the final few months of pregnancy. This is especially critical as several such postnatal behaviors are subject to strong social norms such as feeding newborns with jaggery (as opposed to exclusive breastfeeding) or applying substances to the cord that can cause infections. It is unclear to what extent the ASHA alone can overcome such norms, and most likely a government-driven effort is needed as was done to establish facility delivery as a norm.^24^

Previous research often recommends that ASHAs improve their communication skills,^10^ but specific recommendations are lacking or based on small qualitative samples. ASHA training gives general communication tips but lacks specific recommendations to influence households on key behaviors and rarely mentions involving other stakeholders within the household. We found that many ASHAs did not use many behavior-change messages that could have been effective in driving higher rates of ANC visits and ID. They need to learn when to apply different messages and which individuals in the households should be counseled to achieve greatest impact (which may or may not be the primary decision maker). Our findings suggest that on average, the ASHA should target the pregnant woman for messaging about IFA and checkups, the husband and MIL for ID, and the MIL regarding postnatal care behaviors. Cultural barriers can inhibit the ASHA from talking to the husband or MIL,^10^ so supervision and peer-learning structures may support ASHAs in solving these challenges. A study on improving communication by the ASHA around pneumonia is under way,^25^ and our hope is that such initiatives will be expanded to maternal and neonatal domains. Our findings could also inform future editions of the ASHA training manuals, Sangini (supervisor) training, and technology-enabled job aids for ASHAs.

Communication and behavior change are core skills for ASHAs but receive insufficient attention during training.

Potential guidelines that might result from synthesizing these antenatal results include: the ASHA should visit a woman at her home as soon as possible after learning she is pregnant and 4–6 times over the course of the pregnancy.^16^ During these visits, the ASHA should focus on the woman regarding IFA and antenatal checkups and use a range of messaging strategies such as emphasizing that checkups are now common, doctors recommend them, and that without checkups the woman is at risk of serious undetected complications. During these visits, she should also counsel the husband and MIL on the merits of ID, emphasizing the dangers of home delivery and the risk to life in case of complications. The ASHA should further advise the woman and MIL regarding neonatal care behaviors to increase the likelihood of recommended behaviors in the hours after birth. Although the details might be different for other CHW programs, similar analyses performed on other CHW cadres across the world could help identify opportunities for improvement.

The ASHA’s presence at births in public and private facilities was only weakly associated with targeted health behaviors and respectful care by staff (which many women in LMICs do not receive).^14^ There are several plausible mechanisms behind the observed associations: for example, adoption of recommended breastfeeding behavior may have been due to ASHA counseling, direct support, interaction with facility staff, or other reasons. In the case of respectful care, the ASHA did not wield direct power over the nurses or doctors at the facility. However, the ASHA was familiar with these individuals, was part of the health system, and could therefore have facilitated a positive relationship between community and facility staff. The duration of the ASHA’s presence at birth did not seem to affect any health behavior, throwing into question whether the many hours spent after accompanying the woman to hospital are an optimal use of her time. An important caveat is that we did not investigate other potentially positive effects of the ASHA’s presence at facilities, nor take into account the time spent at the facility waiting for a sign-off for her incentives or for transport home. Nevertheless, the reasons and potential benefits of her presence should be explored further to determine how useful it is for her to spend such long periods of time at facilities. The ASHA might use some of this time better making home visits to which she dedicates less than half as much time as facility visits and indeed less time than she spends on paperwork.

The ASHA’s influence seems more substantial at home deliveries, where the only other outside support usually comes from a traditional birth attendant. The disconnect between the ASHA’s training—which requires her presence for home births—and the low actual rate of presence can partly be explained by the fact that she receives no financial incentive for attending. A direct incentive would contradict the government’s drive for ID, but more creative ways of encouraging the ASHA’s presence could be considered (e.g., an incentive to accompany home-delivered babies for checkups at facilities soon after delivery, which would encourage an ongoing relationship between ASHA and household).

Past work has extensively studied CHW knowledge levels as a proxy for performance.^26^^–^^29^ We were able to show an association between ASHA lack of knowledge of delayed bathing and actual timing of first bathing by households. Therefore, we suggest that guidelines for ASHAs and their supervisors be more explicit about delayed bathing, since the current guidelines are ambiguous, advising only not to bathe the baby “immediately after birth.” The World Health Organization advocates a delay of at least 24 hours,^30^ and the government of UP sets 72+ hours as a target.

These findings from UP add to a substantial body of evidence across the developing world suggesting that CHWs can play an important role in safeguarding the health of women and their newborns,^4^^,^^6^^,^^20^^,^^21^^,^^31^^–^^33^ especially those in the poorest parts of society.^22^ Their position on the frontlines provides a bridge especially for the rural poor into the formal healthcare system. However, it is often hard to understand **why** particular actions predict good health outcomes or why certain actions seem ineffectual. Our findings show that local sociocultural factors such as the decision dynamics of households and common false beliefs about neonatal care should inform how the CHW communicates. It is noteworthy that a review found that of 31 training resource packages for CHWs across low- and middle-income countries, more than half contained only materials that were not tailored to the local context.^34^ Other CHW programs should consider the extent to which our findings might apply to their context and update their guidelines and incentive structures to reflect the optimal number and timing of visits, how CHWs are spending their time, and communication strategies. If resources allow, CHW programs should perform their own research into these questions, borrowing from the multiactor approach presented in this article.

Most research on community health workers reports what is happening; understanding why will be critical to refining these programs.

### Limitations

This study has several limitations. First, the cross-sectional data does not allow us to assess the extent to which CHW actions directly caused behaviors. To mitigate this issue we added covariates capturing the CHW’s actions other than the action being estimated (e.g., to model the association between postnatal visits and exclusive breastfeeding, the model controlled for presence or absence of the ASHA at birth as well as number of antenatal visits), reducing though not eliminating confounding.^35^ Empirical studies in the field could test for any causal nature to the associations uncovered here, for example, comparing 2 regimens of antenatal home visits with differing number of visits, or randomly assigning messages to ASHAs to use during home visits to test which ASHA actions are most likely to raise recommended health behavior rates. Second, the household behaviors are only partially under the control of the CHW. For example, a 24-hour facility stay after the birth, which was entirely unrelated to CHW actions, might be determined by the number of beds in a facility and availability of food. Similarly, the ability to get to facilities when labor starts depended on transport being available. ASHAs are only 1 piece of the puzzle of improving health and outcomes,^6^ and care should be taken not to shoulder ASHAs with responsibilities where they cannot exert control. In UP especially, ASHAs are often of a lower caste, meaning they might be restricted in what households they can visit and who they can counsel within the household. Others have not found a relationship between the mother’s caste and the number of ASHA visits received,^21^ nor was the relationship between ASHA support and health behaviors modified by caste.^21^ Nonetheless, given the known social dynamics around caste, further investigation potentially using the data collected here is warranted.

## CONCLUSION

The mechanisms by which CHWs can contribute to better health for their communities are complex. Each household behavior faces its own set of contextual and perceptual drivers (a framework to comprehensively enumerate such factors can be found in Engl et al.^36^), and progress hinges on understanding these. Strengthening CHW programs worldwide requires going beyond descriptive reports to understand the causal pathways by which the CHW achieves impact. As we continue to ask more of CHWs, we must provide them with the tools and skills to manage an ever-increasing diversity of tasks.

## Supplementary Material

20-00027-Supplement_3.pdf

20-00027-Supplement_5.pdf

20-00027-Supplement_4.pdf

20-00027-Supplement_2.pdf

20-00027-Supplement_1.pdf

## Notes

**Peer Reviewed**

**First published online:** September 22, 2020
