# Supplementary material for: Bringing Greater Precision to Interactions Between Community Health Workers and Households to Improve Maternal and Newborn Health Outcomes in India
Source: Glob Health Sci Pract. 2020 Sep 30;8(3):358–71. doi: 10.9745/GHSP-D-20-00027 (PMC7541124; doi:10.9745/GHSP-D-20-00027)
Supplement: 20-00027-Supplement_3.pdf [file 20-00027-Supplement_3.pdf]

Supplement to: Smittenaar P, Ramesh BM, Jain M, et al. Bringing greater precision to interactions between community health workers and households to improve maternal and newborn health outcomes. Glob Health Sci Pract. 2020;8(3). https://doi.org/10.9745/GHSP-D-20-200027

Supplement 3. Sample Descriptive Statistics

The final sample included 5469 women, 3064 husbands, and 3626 mothers-in-law. The demographics for the sample are described in Table S1, corrected for oversampling of high-priority districts. The sample is representative of the rural population in this region of India, consisting of a majority Hindu population, with low education levels, living in joint or nuclear households. A substantial minority (20%) has no electricity available in their house. More boys than girls are born, which is commonly observed[1].

Supllemental Table. Description of Household Demographics[a]

**Variable**	**Value**
Number of respondents	Women: 5469; husbands: 3064; mothers-in-law: 3626
Age (mean (SD) years)	Women: 25 (4); husbands: 29 (6); mothers-in-law: 52 (10)
Education (mean (SD) years)	Women: 6 (5); husbands: 8 (5); mothers-in-law: 1 (2)
Religion	84.2% Hindu, 15.7% Muslim, 0.1% other
Caste	27% Scheduled caste, 3% Scheduled tribe, 57% Other backward class, 12% none of the above
Parity of woman (including recent birth)	1 birth: 35%, 2 births: 27%, 3 births: 18%, 4 births: 10%, 5+ births: 10%
Household type	joint: 60%; nuclear: 34%; extended: 3%; with maternal parents: 3%
Electricity in house	80%
Perinatal death (died before 7 days old)	150 out of 5469; UP-weighted proportion 2.7%; 27 per 1000 births
Gender of baby	boy: 52.2%, girl: 47.8%

[a] Descriptive statistics are representative of rural Uttar Pradesh, India

[1] Jha P, Kesler MA, Kumar R, et al. Trends in selective abortions of girls in India: analysis of nationally representative birth histories from 1990 to 2005 and census data from 1991 to 2011. The Lancet 2011;377(9781):1921-28. doi: 10.1016/S0140-6736(11)60649-1
