# Supplementary material for: Bringing Greater Precision to Interactions Between Community Health Workers and Households to Improve Maternal and Newborn Health Outcomes in India
Source: Glob Health Sci Pract. 2020 Sep 30;8(3):358–71. doi: 10.9745/GHSP-D-20-00027 (PMC7541124; doi:10.9745/GHSP-D-20-00027)

Supplement to: Smittenaar P, Ramesh BM, Jain M, et al. Bringing greater precision to interactions between community health workers and households to improve maternal and newborn health outcomes. Glob Health Sci Pract. 2020;8(3). https://doi.org/10.9745/GHSP-D-20-200027

Supplement 5. CHW Counseling of Households – Frequency

Figures 3A and 5B show the association between talking to a particular member of household, and the behavior of that household. Supplemental Figure 2 shows how often each member reports receiving such advice. Ideally, the CHW focuses her efforts on the members of households who are in charge of the respective health decision. In practice, we observe that the woman is most often targeted, followed by the mother-in-law, and finally the husband is least often counseled. Note these percentages include only households which had all 3 members interviewed for this study (n = 2441 households), so there is no bias in respondent availability.

Supplemental Figure 2. Rate of receiving counseling on each topic by household member. Corrected for oversampling of high priority areas.

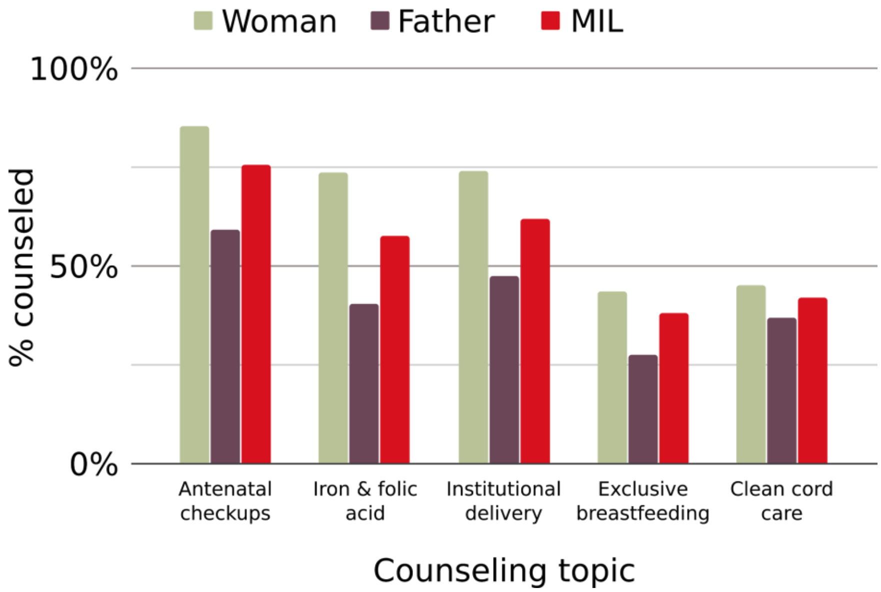

Supplement: 20-00027-Supplement_5.pdf [file 20-00027-Supplement_5.pdf]
