# Supplementary material for: Bringing Greater Precision to Interactions Between Community Health Workers and Households to Improve Maternal and Newborn Health Outcomes in India
Source: Glob Health Sci Pract. 2020 Sep 30;8(3):358–71. doi: 10.9745/GHSP-D-20-00027 (PMC7541124; doi:10.9745/GHSP-D-20-00027)

Supplement to: Smittenaar P, Ramesh BM, Jain M, et al. Bringing greater precision to interactions between community health workers and households to improve maternal and newborn health outcomes. Glob Health Sci Pract. 2020;8(3). https://doi.org/10.9745/GHSP-D-20-200027

Supplement 4. Community Health Worker Time Expenditure

Supplemental Figure 1. Average Time Expenditure Per Month as Self-Reported by Community Health Workers

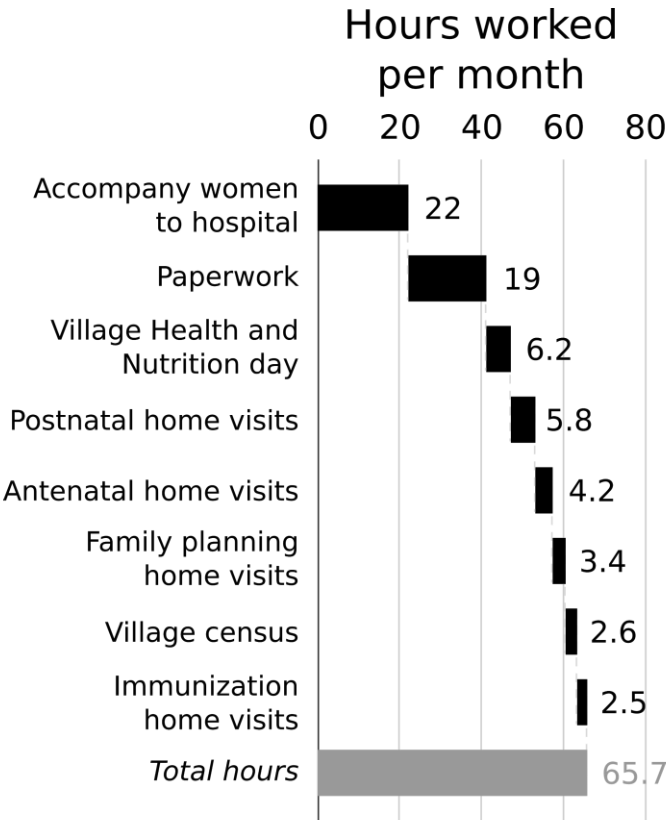

Supplement: 20-00027-Supplement_4.pdf [file 20-00027-Supplement_4.pdf]
