# Supplementary material for: Bringing Greater Precision to Interactions Between Community Health Workers and Households to Improve Maternal and Newborn Health Outcomes in India
Source: Glob Health Sci Pract. 2020 Sep 30;8(3):358–71. doi: 10.9745/GHSP-D-20-00027 (PMC7541124; doi:10.9745/GHSP-D-20-00027)
Supplement: 20-00027-Supplement_2.pdf [file 20-00027-Supplement_2.pdf]

Supplement to: Smittenaar P, Ramesh BM, Jain M, et al. Bringing greater precision to interactions between community health workers and households to improve maternal and newborn health outcomes. Glob Health Sci Pract. 2020;8(3). https://doi.org/10.9745/GHSP-D-20-200027

Supplement 2. Covariate Strategy and Multiple Comparisons

Reducing confounding of relationships through covariates

A central focus of this work was to understand how specific actions by the CHW relate to household behaviors, over and above the other actions a CHW is performing. This is challenging as CHW actions correlate with one another: a household visited more often during pregnancy is also more likely to have the CHW accompany for delivery, and to have the CHW visit in the first week after birth. If left uncontrolled for, virtually each CHW action would seem to be positively associated with each household behavior. The approach applied here was to add covariates capturing the confounding variables. Such a covariate strategy does not perfectly control for confounding[1], but does bring us closer to the specific relationship between individual actions available to the CHW and resulting household behaviors.

We added CHW actions as covariates to each regression as appropriate. For example, when predicting postnatal cord care based on number of antenatal CHW visits, we add covariates for everything the CHW did *outside the antenatal period*: in this case her presence during delivery as well as visits made during the postnatal period. A second example is when predicting exclusive breastfeeding based on number of postnatal CHW visits: we added covariates for everything the CHW did outside the postnatal period, in this case her visits during pregnancy and her presence at delivery.

Multiple comparisons

The analysis consisted of many statistical tests, and it therefore suffers from the multiple comparisons issue. However, the tests were by no means independent, so it is not straightforward to implement an appropriate correction. Our solution is first to acknowledge that these analyses were exploratory rather than confirmatory. Second, we have taken an approach of looking for converging evidence. For example, we test CHW visits by comparing the benefit of any vs no visits, and then perform an orthogonal statistical test by looking for a dose-response effect in only those participants with any visit. If a CHW activity comes out positive in both tests, it is very likely to be a real association rather than a false positive. Third, we visualize adjusted means (also known as estimated marginal means) to assess the magnitude of any association to interpret its relevance. Finally, we qualify the description and interpretation of results by the level of significance, cautioning as appropriate for marginally significant findings.

[1] Westfall J, Yarkoni T. Statistically Controlling for Confounding Constructs Is Harder than You Think. PLoS One 2016;11(3):e0152719. doi: 10.1371/journal.pone.0152719 [published Online First: 2016/04/01]
