# Supplementary material for: Bringing Greater Precision to Interactions Between Community Health Workers and Households to Improve Maternal and Newborn Health Outcomes in India
Source: Glob Health Sci Pract. 2020 Sep 30;8(3):358–71. doi: 10.9745/GHSP-D-20-00027 (PMC7541124; doi:10.9745/GHSP-D-20-00027)
Supplement: 20-00027-Supplement_1.pdf [file 20-00027-Supplement_1.pdf]

Supplement to: Smittenaar P, Ramesh BM, Jain M, et al. Bringing greater precision to interactions between community health workers and households to improve maternal and newborn health outcomes. Glob Health Sci Pract. 2020;8(3). https://doi.org/10.9745/GHSP-D-20-200027

# Supplement 1. Questionnaire Details

The structured surveys were programmed on mobile tablet devices and the interviewer entered responses. Female interviewers collected data from household members, and the CHW was interviewed by an interviewer of either gender. The questionnaire was designed based on qualitative interviews that identified a complex mix of factors important for health decisions. The surveys were highly detailed to capture not only actions but also beliefs, fears, risk perceptions, social pressure, and any other relevant contextual and perceptual drivers of key behaviors[1]. For this reason, the median duration was 1 hour 40 minutes for the household survey, and 2 hours for the CHW survey. Survey fatigue and participant engagement were tracked using surveyor ratings at the end of the interview.

The survey for recently delivered women, husbands, and mothers-in-law was divided into the following sections: 1) 83 questions on pregnancy, including health behaviors during last pregnancy, timing and location of checkups, social support, timing and intensity of visits and advice from CHW, trust in healthcare workers, beliefs and knowledge about good practices, barriers to health behaviors; 2) 132 questions on delivery, including history of deliveries, birth planning, events during birth, interactions with staff, barriers to institutional delivery, trust in staff, opinion of available services and infrastructure, quality of care including respectful care, CHW support at delivery, risk perception, opinion on private versus public facilities, extensive detail on finances (each individual expense, planning, loans), awareness and opinion of government incentive; 3) 72 questions on postnatal care, including behaviors and beliefs around breastfeeding and cord care and delayed bathing and kangaroo care, social support, CHW support, opinion of CHW, risk perception, checkups; 4) 43 questions on personality and household dynamics; 5) 76 questions on family planning, including methods used and using, beliefs about methods, barriers to use (embarrassment, cost), sources of supplies, desired incentives from government, perceived risk and harm; 6) 41 questions on sociodemographics, including age, religion, caste, household composition, domestic violence, income, media consumption, and proxies for wealth (e.g. electricity, material of house, land owned, vehicles). Finally, the interviewer noted down 12 contextual factors, including the location of the house, interviewee responsiveness, subjective understanding of questions, subjective motivation of respondent, and privacy of interview.

This paper primarily uses responses from the recently delivered woman to associate CHW actions with health behaviors. We also draw on information from the CHW survey. This survey consisted of a comprehensive assessment of the CHW's beliefs, actions, opinions, knowledge, and skills for every aspect of maternal and neonatal care as well as family planning. The CHW's work log ("Village Health Index Register") and sociodemographics were also captured. The CHW was assessed on her clinical knowledge, which is used in analyses in this paper. We also presented the CHW with a list of 7 messages that might be used to convince women to attend checkups, and 7 messages to promote ID. The CHW indicated which messages she had ever used in the

[1] Engl E, Sgaier S. CUBES: A practical toolkit to measure enablers and barriers to behavior for effective intervention design [version 1; peer review: 2 approved, 1 approved with reservations]. Gates Open Research 2019;3(886) doi: 10.12688/gatesopenres.12923.1

past. She was then asked to rank-order the messages she had used, based on the question, "If you can pick only one thing to say that has most impact on households, which one will you pick?". This allowed us to determine the "preferred message" for each CHW for influencing households to attend checkups and go for institutional delivery, respectively. We only report on antenatal communication strategies, as no such questions were asked about her strategies around improving postnatal care.

We also asked the CHW about how she spends her time. The CHW indicated the number of times she performs a particular action, and the amount of time it takes her on average, for each of the following: antenatal home visits per month, postnatal home visits per month, accompanying women to hospital for birth or checkups per month, home visits for family planning per month, home visits for child immunization per month, attendance at VHND per year, taking village census per year, and hours on paperwork per month. We then calculated the perceived average number of hours worked on each task per month as well as total hours worked, and from this the share of each activity. Note we did not include some of her other work-related activities such as meetings with her supervisor or attendance to other tasks unrelated to maternal, neonatal, and child health.

The interviewer had visual aids to improve understanding, such as cards illustrating certain care actions. Answers were captured as multiple choice, rating scales, numeric answers, free recall, recognition, and in rare cases as free text.
